# Brainstem infarction in a patient with internal carotid dissection and persistent trigeminal artery: a case report

**DOI:** 10.1186/1471-2342-10-14

**Published:** 2010-07-02

**Authors:** Daniela Iancu, Rene Anxionnat, Serge Bracard

**Affiliations:** 1 Department of Neuroradiology, CHU Nancy, Nancy, 54035, France; 2 Department of Radiology, Section of Neuroradiology, University of Manitoba, Health Sciences Centre, 820 Sherbrook St, Winnipeg, Manitoba R3A 1R9, Canada

## Abstract

**Background:**

The primitive trigeminal artery (PTA) is the most commonly described fetal anastomosis between the carotid and vertebrobasilar circulations.

**Case presentation:**

We report a 42-year-old patient presenting with internal carotid dissection, and imaging features of brainstem infarction.

**Conclusion:**

Based on the imaging studies we presume occlusive carotid dissection with extensive thrombosis within a persistent trigeminal artery as the cause of this brainstem ischemia.

## Background

Several fetal anastomoses have been described between the carotid and vertebrobasilar circulations. These anastomoses regress while the P1 segments develop, but they can occasionally persist in adult age [1]. The primitive trigeminal artery (PTA) is the most common of them representing about 85% of cases with prevalence between 0.1% and 0.76% [2].

We report a patient with brainstem infarction caused by a persistent PTA thrombosis secondary to occlusive dissection of the homolateral internal carotid artery (ICA).

### Case presentation

A 42-year-old woman presented with right-side motor deficit and dysarthria. She experienced diffuse headaches, regressive episodes of ill-defined visual disturbance and right-side numbness the previous day. She reported osteopathic cervical manipulations in the previous week.

Neurologic examination revealed right-sided hemiparesis, hypoesthesia, central facial palsy and dysarthria. No Horner's sign, cranial nerve palsy, abnormal cardiac or carotid bruit were found.

**Figure 1:**
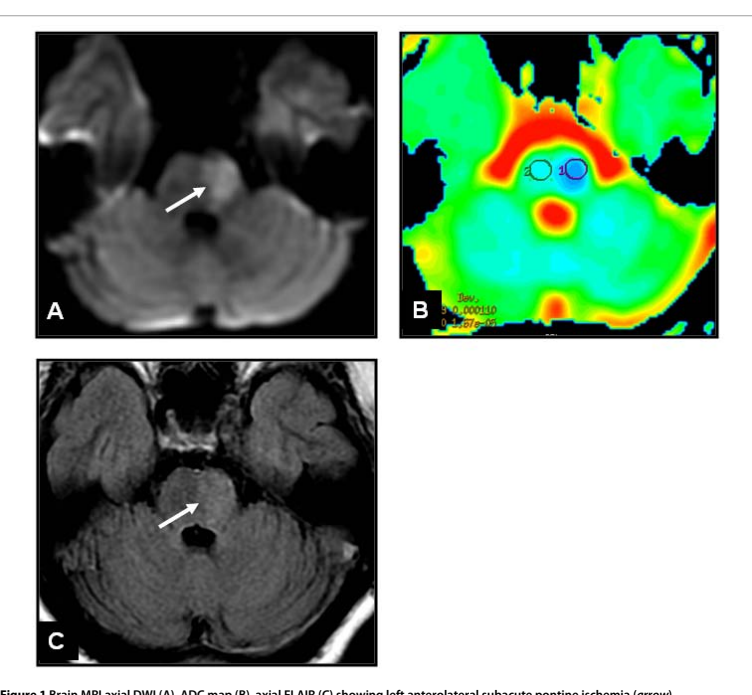
**Brain MRI axial DWI (A), ADC map (B), axial FLAIR (C) showing left anterolateral subacute pontine ischemia (*arrow*)**.

**Figure 2:**
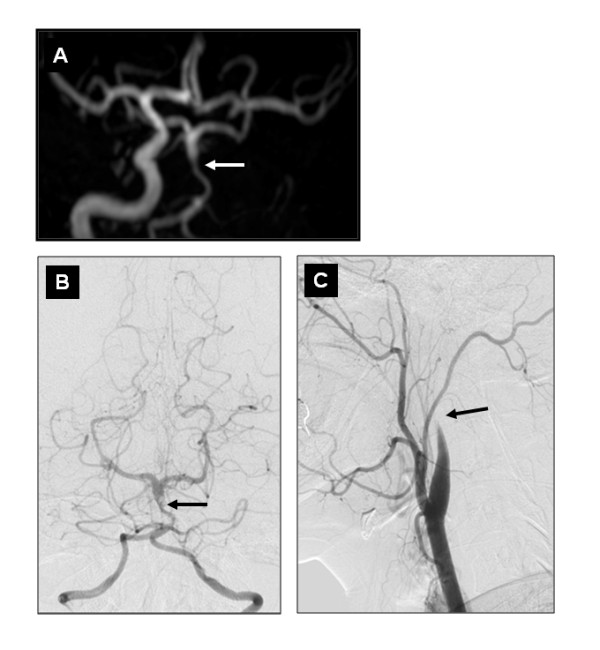
**3D-TOF MRA and left-VA DSA**. (A) 3D-TOF MRA revealing left-ICA occlusion with good cross flow in the left-MCA and ACA and hypoplastic aspect of both VA and proximal BA (*arrow*) usually seen in persistent PTA. (B) Left VA DSA confirming small VA, proximal BA and localized intravascular filling defect on the mid basilar segment proximal to the SCA origin, corresponding to thrombus extension from the PTA (*arrow*). (C) Left common carotid artery DSA showing flamed-shaped occlusion of the left ICA, characteristic of occlusive dissection (*arrow*).

**Figure 3:**
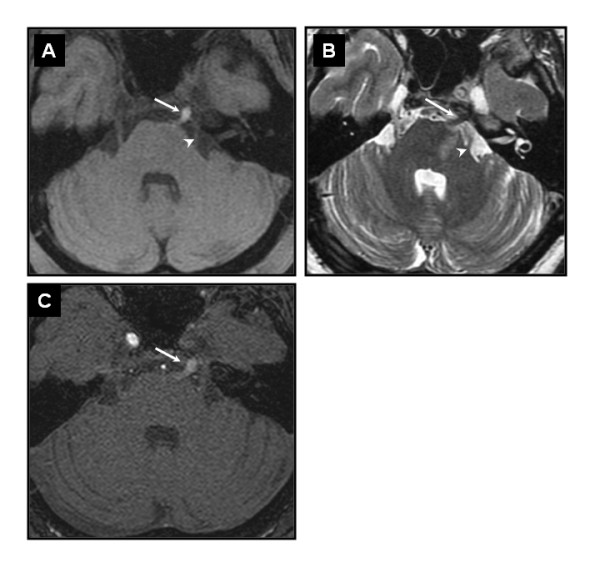
**Thin axial sections MRI**. Axial T1WI with fat-saturation (A), T2WI (B) and axial view of 3D-TOF MRA (C) showing the thrombus in the persistent PTA (*arrow*) appearing hyperintense in T1WI, hypointense on T2WI, medial to the cisternal portion of the trigeminal nerve (*arrowhead*).

Magnetic resonance imaging (MRI) revealed left anterolateral pontine infarction (Figures 1A, B, and 1C). No acute infarction was seen in the left-ICA territory. Three-dimensional time-of-flight MRA (3D-TOF MRA) showed occlusion of the left ICA and hypoplastic vertebral (VA) and proximal basilar (BA) arteries (Figure 2A). The left hemispheric supply was provided by the right-ICA via anterior communicating artery. Additional thin axial sections T2 and T1WI [3] were unable to demonstrate dissections on vertebrobasilar system. However, a well defined 5mm structure, hyperintense on T1WI with fat-saturation and hypointense on T2WI was seen within the prepontine cistern via Meckel's cave with a similar course to the trigeminal nerve, which was identified separately. In view of its location and orientation we presumed that this structure corresponded to a thrombosed persistent PTA and concluded that the brainstem stroke was due to an extensive thrombosis caused by occlusive ICA dissection via the PTA (Figure 3).

A digital subtraction angiography (DSA) showed irregular localized filling defect within the distal hypoplastic BA (Figure 2B) and a flame-shaped occlusion of the left ICA which is characteristic of occlusive dissection (Figure 2C).

Further investigation did not reveal concomitant cardiac or coagulation disorders. Intravenous Heparin was initiated and the patient was discharged 3 weeks later with residual motor deficit. MRA follow-up showed persistent ICA and PTA occlusion.

## Discussion

Persistent PTA is often associated with intracranial aneurysms, arteriovenous malformations, carotid cavernous fistulas, Moyamoya and cerebellar hemangioblastoma [4-7]. Its clinical significance is usually uncertain but presentation may include cranial nerve dysfunction or subarachnoid haemorrhage [4-7].

Very few cases of brainstem or occipital infarction due to embolism from the ICA stenosis via persistent PTA have been reported [8,9]. To our knowledge, this is the only case report documenting a persistent PTA thrombosis responsible for a brainstem infarction. The diagnosis was difficult since flow was completely absent in the PTA even on DSA. One clue was the diminutive aspect of both VA and proximal BA, usually found in persistent PTA. Moreover, concomitant dissection of the ICA and BA would have been unlikely.

The PTA courses from the ICA cavernous segment to the BA between the origins of the anterior inferior cerebellar arteries (AICA) and the SCA. It usually follows the trigeminal nerve in the cisternal part and Meckel's cave with some anatomic variants [1]. The localization of the thrombosis on MRI appears consistent with the known anatomy. Embryologically, the PTA is a metameric artery arising from the first aortic arch, supplying central and peripheral nervous structures. It follows the trigeminal nerve, ending in a plexus at the trigeminal ganglion. Later on, PTA is the most important supply of the posterior structures but ultimately it regresses usually at the carotid edge [1]. The pontine artery could give an accessory branch to the trigeminal ganglion but its main territory is the protuberance [1]. In this case the anterolateral pontine infarction corresponds to this territory.

Stroke secondary to cervical ICA dissection generally involves embolic mechanisms instead of hypoperfusion. Since dissection rarely extends beyond the petrous segment of the ICA [10], we propose that extensive thrombosis, due to the dissection, is the mechanism that occluded the intracavernous carotid segment and the PTA. The DSA aspect showing small VA and proximal BA with localized endovascular filling defect is suggestive of pre-existent PTA pattern with a distal extension of the thrombosis into the PTA and BA.

## Conclusion

This is a very rare case of MRI documented persistent PTA thrombosis responsible for brainstem infarction. In patients presenting with brainstem ischemia associated with occlusion or stenosis of the homolateral ICA, persistent PTA should be considered.

## Consent

Written informed consent was obtained from the patient for publication of this case report and any accompanying images. A copy of the written consent was provided to the editorial office of this journal.

## Competing interests

The authors declare that they have no competing interests.

## Authors' contributions

DI performed the literature search and compiled data presented in this report. RA provided the expertise for selective imaging and contributed to the diagnosis. SB provided intellectual input and critically revised the manuscript. All authors read and approved the final manuscript.

## Pre-publication history

The pre-publication history for this paper can be accessed here:

http://www.biomedcentral.com/1471-2342/10/14/prepub
